# Turn-on Type Chemical Sensing of Vitamin K4 by Fluorene Dendrimers with Naphthalene Segments

**DOI:** 10.3390/molecules19044135

**Published:** 2014-04-02

**Authors:** Naoya Adachi, Hiroki Sugiyama, Masafumi Arai, Hideo Ogawa

**Affiliations:** 1 Division of Liberal Arts, School of Science and Engineering, Tokyo Denki University, Hatoyama, Hiki-gun, Saitama 350-0394, Japan; 2 Division of Science, School of Science and Engineering, Tokyo Denki University, Hatoyama, Hiki-gun, Saitama 350-0394, Japan

**Keywords:** dendrimer, chemical sensor, vitamin K4, fluorescence turn-on

## Abstract

G1 and G2 fluorene dendrimers with naphthalene termini were synthesized as a fluorescence turn-on type chemical sensor for vitamin K4. The fluorene dendrimers were prepared by Williamson ether reaction between the fluorene core with dihydroxy groups and dendritic naphthalene segments with methylene chloride by a convergent method. We investigated the relationship between the dendrimer generation and vitamin K4 recognition of fluorene dendrimer with naphthalene termini in CHCl_3_. Addition of vitamin K4 enhanced the fluorescence intensity of the fluorene dendrimer. Especially, the G2 fluorene dendrimer was found to be an effective chemical sensor for vitamin K4 and better than the G1 fluorene dendrimer.

## 1. Introduction

Vitamins are organic nutrients in which all carbohydrates, proteins, mineral, and lipids have been removed [1,2]. Vitamins, one of the most important nutrients, must be ingested (preferably as a constituent of food but alternatively as a dietary supplement) because most are not produced by humans *in vivo* [3,4]. A lack of vitamins can cause vitamin deficiencies that lead to diseases such as beriberi, scurvy, and pellagra [5]. To aid in disease prevention, vitamin requirements have been determined on the basis of daily intake and excretion [6,7,8]. A number of research groups have reported that vitamins prevent cardiovascular diseases, cancer, osteoporosis, and type 2 diabetes [5,9,10]. Vitamins are categorized as water-soluble vitamins such as vitamins B and C and lipid-soluble vitamins such as vitamins A, D, E, and K. Vitamin K is a lipid soluble vitamin that has the important functions of inducing blood coagulation and preventing arterial calcification [5,11]. Thus, the importance of vitamin K is rapidly expanding in the fields of medicine and healthcare [12,13]. An accurate measurement technique is necessary to determine the concentration and quantity of vitamin K present. Typically, vitamin K is detected by high-performance liquid chromatographic and electrochemical methods [14,15,16]. However, these methods have low selectivity and are irreversible and time-consuming, so another simple method that has high selectivity and high sensitivity is desired.

Dendrimers possess a symmetrical structure that usually three-dimensional and globular, and composed of a central core with multi branched side chains called dendrons [17]. Various low molecular weight compounds can be introduced into the nano-space of the high-generation dendrons of a dendrimer; such compounds include metal nanoparticles, fluorescent dyes, drugs, small biomolecules, proteins, and ions [18,19]. These dendrimer materials are expected to find application in organic light-emitting diodes, solar cells, drug delivery systems, catalysts, and chemical/biological sensors [20,21,22]. In particular, types of highly-fluorescence-intensity dendrimers have been used as the analytical materials in chemical sensors, fluorescence markers, and biological sensors. Typically, chemical sensors based on dendrimer fluorescence have been of the turn-off type, using fluorescence quenching of the dendrimer to detect target compounds [22,23,24]. In contrast, turn-on type chemical sensors use fluorescence enhancement to detect of target compounds [25,26]. Kawakami *et al.* reported the use of a dendrimer having naphthalene units as a fluorescence turn-on type chemical sensor for metal ions [27,28]. In this paper, we report a fluorescence turn-on type chemical sensor for vitamin K4 in chloroform using a fluorene dendrimer with G2 naphthalene dendrons. Fluorescence spectra revealed that the synthesized fluorene dendrimer acted as a high-sensitivity turn-on type chemical sensor for vitamin K4. Furthermore, our results offer potential opportunities for future applications of simple dendrimer-based turn-on type chemical sensors.

## 2. Results and Discussion

### 2.1.Synthesis of G1 and G2 Dendrimers

**Scheme 1:**
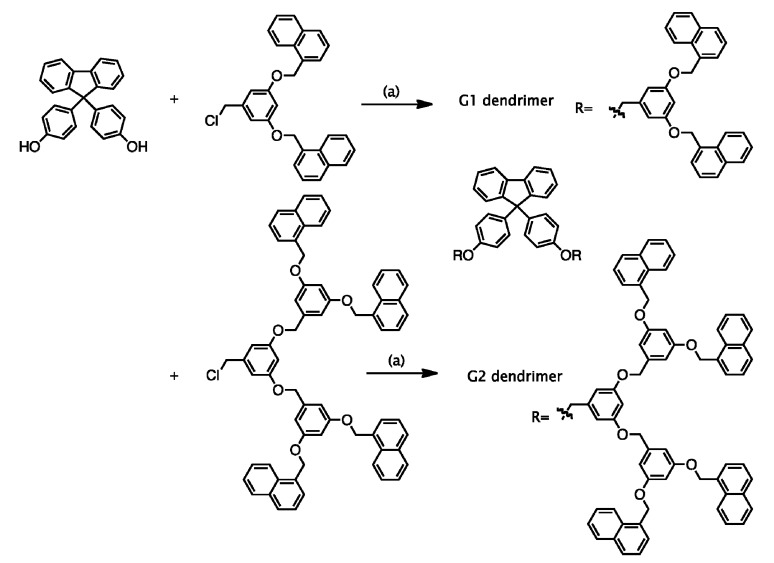
Chemical structure of G1 and G2 dendrimers.

The synthetic scheme for the first- and second-generation (G1 and G2) fluorene dendrimers with naphthalene termini is shown in Scheme 1. A convergent method was applied to synthesize the G1 and G2 fluorene dendrimers [29]. The G1 and G2 benzyl ether dendrons with naphthalene termini were synthesized by the convergent method developed by Hawker and Fréchet [30] using 3,5-dihydroxybenzoic acid methyl ester and naphthyl chloride as starting materials. The reduction by LiAlH_4_ and then chlorination of SOCl_2_ of the G1 and G2 dendrons gave the corresponding monomers G1-CH_2_Cl and G2-CH_2_Cl in 89% and 86% yields, respectively. The G1 and G2 fluorene dendrimers with naphthalene termini were prepared by Williamson ether reactions between 4,4'-(9-fluorenylidene)diphenol with the synthesized G1- and G2-CH_2_Cl_2_ dendrons in 40% and 71% yields, respectively. ^1^H-NMR spectroscopy confirmed the successful synthesis of the G1 and G2 dendrimers. The synthesized G1 and G2 dendrimers exhibited good solubility in common organic solvents such as THF, CHCl_3_, CH_2_Cl_2_, acetonitrile and toluene.

*Reagents and Conditions:* (**a**) K_2_CO_3_, DMF, 60 °C, 24 h.

### 2.2. Optical Properties

Optical properties of the G1 and G2 dendrimers were studied by UV-vis and fluorescence measurements and are summarized in Table 1.

**Table 1:** Optical properties of G1 and G2 dendrimer.

	Abs λ (nm)	ε (M^−1^ cm^−1^)	Emission λ (nm)
G1 dendrimer	283	60,000	330
G2 dendrimer	283	110,000	330, 440

**Figure 1:**
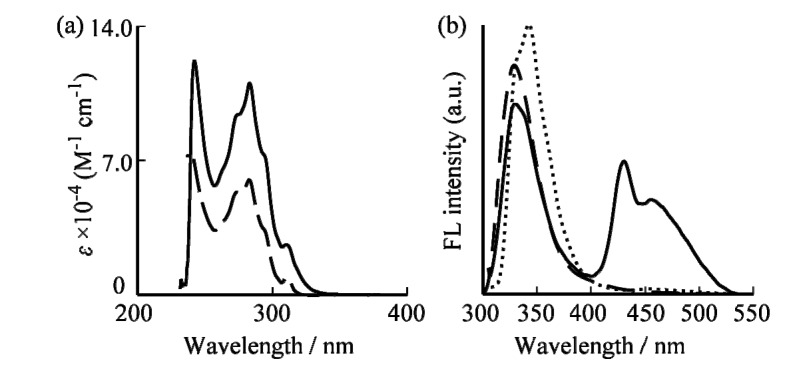
(**a**) UV-vis and (**b**) fluorescence spectra of G1 (dashed line) and G2 (solid line) dendrimers and vitamin K4 (dotted line) in CHCl_3_.

UV-vis absorption spectra of the G1 and G2 dendrimers are shown in Figure 1a Mini-meeitng - BRB. The absorption spectra of G1 and G2 dendrimers were investigated in CHCl_3_ solutions in which the G1 and G2 dendrimer concentrations were both fixed at 1.0 × 10^−5^ M. The G1 and G2 dendrimers displayed a maximum absorption band in the UV region at 283 nm. The maximum molar extinction coefficient at 283 nm, which increases in intensity with the dendrimer generation, can be assigned to the dendrimer branches [31]. With increasing generation, the absorption band edges for G2 and G1 shifted to longer wavelengths. As the dendrimer generation increased, the molar extinction coefficient corresponding to the number of dendron units in G2 dendrimer almost doubled over that of the G1 dendrimer. Thus, the light-harvesting property of the G2 dendrimer will be significantly improved over that of the G1 dendrimer.

The steady-state fluorescence emission spectra of G1 and G2 fluorene dendrimers in CHCl_3_ at room temperature are shown in Figure 1b. As in Figure 1a, the concentration of both G1 and G2 dendrimers was 1.0 × 10^−5^ M. The fluorescence spectrum of the G2 dendrimer was observed at around 330 and 440 nm by excitation at 283 nm. The sharp fluorescence at 330 nm can be assigned to the emission from the naphthalene segments into the dendrimers [31,32,33]. Broad emissions were observed in the longer wavelength region for the G2 dendrimer. This broad fluorescence peak at 440 nm is due to the excited state intramolecular excimer formation between naphthalene units.

### 2.3. UV-Vis and Fluorescence Detection of Vitamin K4

**Figure 2:**
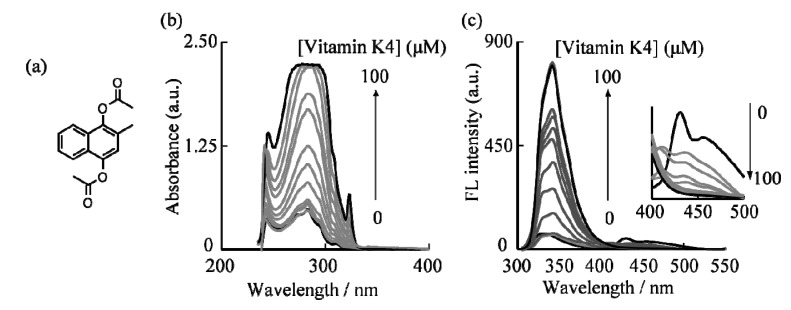
(**a**) Chemical structure of vitamin K4; (**b**) UV-vis and (**c**) fluorescence response of G2 dendrimer upon addition of vitamin K4. The inset shows fluorescence response of G2 dendrimer upon addition of vitamin K4 at 400–500 nm.

The sensing properties of G1 and G2 dendrimers to vitamin K4 (2-methylnaphthalene-1,4-diacetate) (Figure 2a) was assessed in CHCl_3_ solution at a dendrimer concentration of 1.0 × 10^−6^ M at room temperature. The isosbestic points of the dendrimers’ absorption spectra which would prevent nonlinearity of the fluorescence intensity were chosen as the excitation wavelength of the G1 and G2 dendrimers. Figure 2c shows the changes in the fluorescence spectra of the G2 dendrimer in CHCl_3_ solution upon titration of vitamin K4. When vitamin K4 was added to a CHCl_3_ solution of G1 and G2 dendrimers, changes in the intensity and shape of the fluorescence spectra were noted.

To study the UV-vis absorption and fluorescence behavior of G2 dendrimer with vitamin K4, the UV-vis and fluorescence were precisely measured. The changes in the UV-vis absorption spectra of the G2 dendrimer in CHCl_3_ solution upon titration of vitamin K4 are shown in Figure 2b. The absorption band at 242, 283, and 315 nm was increased by addition of vitamin K4. As shown Figure 2c, addition of vitamin K4 caused an increase at 340 nm and a decrease at 440 nm in the fluorescence intensity of the G2 dendrimer.

**Figure 3:**
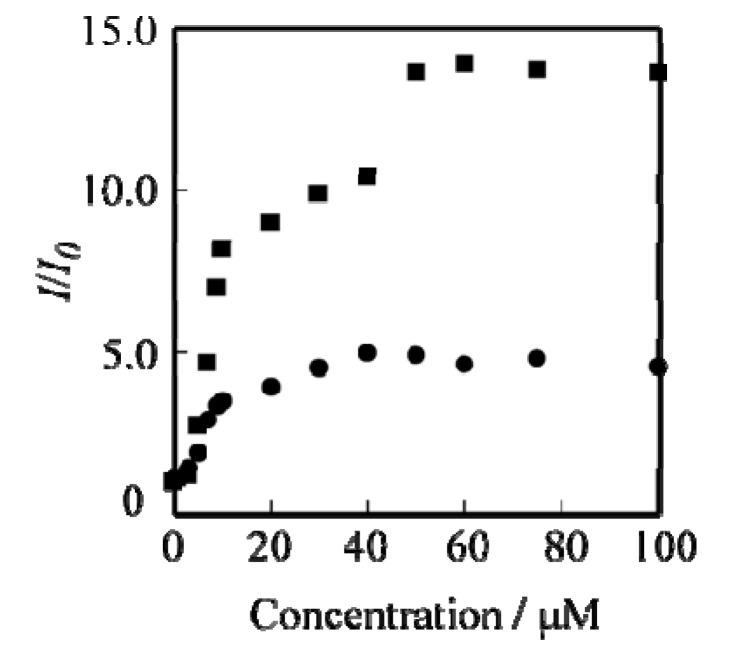
Changes in the fluorescence intensity of G1 (●) and G2 (■) dendrimer as a function of vitamin K4 concentration.

The changes in absorption and fluorescence intensity became constant when the amount of vitamin K4 reached 6.0 × 10^−5^ M, and an approximately 13-fold fluorescence intensity enhancement could be observed (Figure 3). Changes in the fluorescence intensity of G1 and G2 dendrimers at 340 nm by addition of several vitamin K4 concentrations are shown in Figure 3. The changes in the fluorescence intensity (*I*/*I_0_*) have been determined form the ratio of maximum fluorescence intensity (*I*: addition of several concentrations of vitamin K4) at 340 nm and minimum fluorescence intensity (*I_0_*: absence of vitamin K4) at 340 nm [34]. The saturation point of increasing fluorescence intensity in the G1 and G2 dendrimer-vitamin K4 system was reached when the vitamin K4 concentration was 4.0 × 10^−5^ M and 6.0 × 10^−5^ M, respectively. The G2 dendrimer, with its many naphthalene units, is an excellent fluorescent chemical sensor for vitamin K4, much better than the G1 dendrimer. The excimer emission at 440 nm nearly disappeared when the addition of vitamin K4 reached 10 × 10^−6^ M in the G2 dendrimer solution. Disappearance of the excimer emission peak at 440 nm suggested that the naphthalene units can trap vitamin K4 by π–π interactions. The addition of vitamin K4 could lead to about a 13-fold emission intensity enhancement for G2 dendrimer, indicating that the turn-on fluorescence measurement can detect vitamin K4 at the ppm level and can be used as a highly sensitive chemical sensor for vitamin K4. These results demonstrated that the detection limit value of vitamin K4 was 15.5 ppm.

**Scheme 2:**
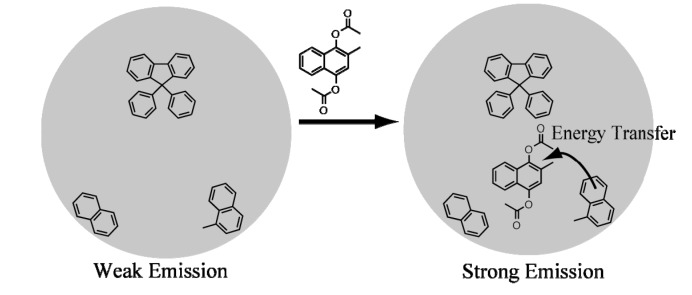
Schematic illustration of G2 fluorene dendrimer-vitamin K4 turn-on fluorescence sensor mechanism.

When vitamin K4 was gradually added to the G1 or G2 dendrimer solution, the excimer emission at 440 nm decreased and monomeric naphthalene emission at 340 nm increased. The fluorescence emission spectrum of vitamin K4 in CHCl_3_ at room temperature is shown in Figure 1b. The observed fluorescence spectrum was overlapped substantially with the dendrimers fluorescence spectrum. These results are consistent with the proposed turn-on type fluorescence mechanism in Scheme 2, which indicates that the intermolecular energy transfer occurred from the dendrimers to the encapsulated vitamin K4 upon addition of vitamin K4. In addition, the energy transfer process in the dendrimers with naphthalene units-vitamin K4 system to the energy transfer that occurred from the dendrimers having naphthalene termini (the energy donor) to the vitamin K4 with ester groups (the energy acceptor). The sensing process of G1 and G2 dendrimers to vitamin K4 was expected that the dendrimers can encapsulate vitamin K4 by π–π interactions between naphthalene units or fluorene core and vitamin K4 and then the fluorescence intensity was increased by the energy transfer from naphthalene units to vitamin K4.

## 3. Experimental

### 3.1. General

All reagents and solvents were purchased from Wako Chemicals (Osaka, Japan), Tokyo Kasei (Tokyo, Japan), or Aldrich (Sigma-Aldrich, Saint-Louis, MO, USA) in analytical grade. All solvents were distilled before each procedure. Ultrapure water of greater than 18.2 MΩ^.^cm was supplied by a Milli-Q system (Merck Millipore, E. Merck, Darmstadt, Germany) and was used throughout all experiments. Column chromatography was performed on a Wakogel C-200. Analytical thin-layer chromatography (TLC) was conducted using Merck 0.25 mm silica gel 60F-coated aluminum plates with fluorescent indicator UV254. NMR spectra were recorded on a Bruker AVANCE 300 FT NMR spectrometer (Bruker Corporation, Billerica, MA, USA) operating at 300.13 MHz for ^1^H in CDCl_3_ as solvent. Chemical shifts were reported relative to internal TMS. UV-Vis spectra were recorded on a Shimadzu UV-1700 spectrometer (Shimazdu, Chiyoda-ku, Japan). Fluorescence spectra were measured on a JASCO FP-6200ST spectrophotometer (JASCO, Hachioji, Japan). All optical measurements were performed at room temperature unless otherwise stated.

### 3.2. Synthesis of G1 and G2 Dendrimers

*G1 dendrimer* [30]. K_2_CO_3_ (0.19 g, 1.35 × 10^−3^ mol) was added to a solution of 4,4'-(9-fluorenylidene)diphenol (23 mg, 6.42 × 10^−5^ mol) and G1-CH_2_Cl (0.13 g, 1.35 × 10^−4^ mol) in 10 mL of distilled DMF. The reaction mixture was stirred for 24 h at 80 °C. After 24 h, the reaction mixture was diluted with CH_2_Cl_2_ (50 mL) and washed with water (3 × 50 mL) and brine (3 × 50 mL). The organic layer was dried over MgSO_4_ and concentrated under vacuum. The pure white solid was obtained by column chromatography (1:1 CH_2_Cl_2_/hexane). Yield: 40%. ^1^H-NMR (CDCl_3_): δ = 8.04 (d, *J* = 8.6 Hz, 4H, Ph), 7.91 (d, *J* = 1.7 Hz, 8H, Ph), 7.77 (d, *J* = 7.6 Hz, 2H, Ph), 7.42 (m, 24H, Ph), 7.14 (d, *J* = 2.1 Hz, 4H, Ph), 6.81 (m, 10H, Ph), 5.39 (s, 8H, -OC*H*_2_-naphthalene), 4.99 (s, 4H, -OC*H*_2_-Ph).

*G2 dendrimer* [30]. The synthetic procedure for G2 dendrimer is similar to that of G1 dendrimer. G2 dendrimer was prepared from 4,4'-(9-fluorenylidene)diphenol and G2-CH_2_Cl. Yield: 71%. ^1^H-NMR (CDCl_3_): δ = 8.03 (d, *J* = 8.6 Hz, 8H, Ph), 7.86 (m, 16H, Ph), 7.73 (d, *J* = 3.7 Hz, 2H, Ph), 7.56 (m, 40H, Ph), 7.10 (d, *J* = 4.1 Hz, 4H, Ph), 6.80 (m, 20H, Ph), 5.45 (s, 16H, -OC*H*_2_-naphthalene), 4.94 (s, 12H, -OC*H*_2_-Ph).

## 4. Conclusions

In conclusion, we have developed a fluorescence turn-on type chemical sensor for vitamin K4 that uses a fluorene dendrimer with naphthalene units. G1 and G2 fluorene dendrimers having naphthalene termini were synthesized by a Williamson ether reaction. The synthesized G2 fluorene dendrimers was highly soluble in various solvents and had high light-harvesting ability. The changes in the shape and intensity of fluorescence spectra that occurred upon vitamin K4 addition were great for the G2 dendrimer. We have also demonstrated that this fluorene dendrimer can be used as a high-sensitivity chemical sensor for vitamin K4. These chemical sensors might be exploited for application of fluorescence markers and liposomes.
